# Immediate Leg Mobilization is Feasible After Catheter Ablation of Atrial Fibrillation Using Large Vascular Access Sheaths (Pulsed Field and Cryoballoon Ablation)

**DOI:** 10.1111/jce.70050

**Published:** 2025-08-06

**Authors:** Riya Sam, Romil Patel, Lavisha Singh, Westby Fisher, Mark Metzl, Jose Nazari, Alex Ro, Hany Demo, Jeremiah Wasserlauf

**Affiliations:** ^1^ Division of Cardiology Endeavor Health System – NorthShore University Health System Glenbrook Illinois USA; ^2^ University of Chicago Pritzker School of Medicine Chicago Illinois USA

**Keywords:** atrial fibrillation, cryoballoon ablation, Perclose Proglide, pulmonary vein isolation, pulsed field ablation, suture‐mediated vascular closure

## Abstract

**Background:**

Vascular recovery from catheter ablation (CA) has traditionally required a period of leg immobilization which can lead to discomfort and prolonged time to discharge.

**Objective:**

The objective of this study was to compare a strategy of immediate leg mobilization (IM) using suture‐mediated closure devices against traditional vascular recovery consisting of figure‐of‐eight suture and 4 h bed rest (BR) after CA of atrial fibrillation (AF) using large vascular access sheaths for cryoballoon ablation (CBA) and pulsed field ablation (PFA).

**Methods:**

Two hundred subjects were retrospectively analyzed: 100 IM and 100 BR. Vascular closure in IM consisted of a single suture‐mediated closure delivered to each of three venotomies. Following the procedure, both legs could be mobilized immediately with no head‐of‐bed restriction. Ambulation was instructed at 1 h, and discharge at 3 h postprocedure. Hemostasis in BR was achieved using figure‐of‐eight sutures and 4 h of BR. The primary endpoint was the incidence of vascular complications. The secondary endpoint was time to discharge.

**Results:**

The mean age was 68.4 ± 11.7 years. In total, 72% of subjects in IM and 5% of the subjects in BR were treated with PFA. There was no difference in vascular complications (1 IM vs. 0 BR, *p* = 0.316). Time to discharge was shorter in IM (4.2 ± 2.6 h vs. 6.0 ± 2.7 h, *p* < 0.05).

**Conclusion:**

Immediate mobilization with suture‐mediated vascular closure following CBA or PFA was associated with no difference in vascular complications compared to 4 h BR and shorter time to discharge. Further studies are needed to illustrate potential benefits to patient comfort and satisfaction.

## 1 Introduction

Atrial fibrillation (AF) is the most common sustained arrhythmia worldwide. Catheter ablation (CA) is supported by a guideline indication for AF in patients refractory or intolerant of antiarrhythmic drugs, and as a first‐line therapy in selected patients [1]. Ablation technologies such as cryoballoon ablation (CBA) or pulsed field ablation (PFA) are widely used and typically use large‐bore venous access. Vascular complications increase with the number and size of sheaths, and when coupled with the use of intraprocedural anticoagulation, can be associated with access site bleeding and hematoma. Current measures to reduce vascular complications include ultrasound‐guided venous access, figure‐of‐eight sutures, manual compression, pressure bandages, and leg immobilization [2]. Suture‐mediated vascular closure (SMVC) systems are feasible for use in electrophysiology procedures and facilitate same‐day discharge [3]. Perclose ProStyle and ProGlide (Abbott, Santa Clara, CA, USA) are one such class of SMVC that are FDA approved for closing femoral venous access sites using 5F to 24F sheaths. For sheath sizes > 14F, at least two devices and preclose technique are required in the labeling. Patients may be able to move freely in bed without head of bed or leg restrictions if the close is successful. Patients who have undergone cardiac arrhythmia treatments with multiple access sites in a single femoral vein of one or both limbs may be ambulated 1 h or more and may be eligible for same‐day discharge 2 h or more after the Perclose ProStyle SMCR procedures based on the judgment of the physician [4]. This study aimed to compare a strategy of immediate leg mobilization (IM) using SMVC versus traditional 4‐h bed rest with figure‐of‐eight suture (BR) after CA of AF using large vascular access sheaths.

## 2 Methods

### 2.1 Study Population

This retrospective study included 100 consecutive patients who underwent CA using CBA or PFA at Endeavor Health System between January 2024 and October 2024 with an IM strategy. IM was adopted by individual operators at their discretion, while others utilized BR. In total, 100 BR cases were identified to form a control group. In order to reach an identical sample size, historical subjects were included in the control group. Therefore, procedures in the BR group took place from January 2023 to April 2024. The IM protocol was adopted in 2024 and coincided with commercial approval and institutional adoption of PFA, therefore, there is a greater proportion of PFA cases in this group. This study was approved by the Endeavor Health System Institutional Review Board with a waiver of consent. Inclusion criteria consisted of age above 18 years and an ECG‐documented diagnosis of AF undergoing the above ablation procedure.

### 2.2 Ablation Procedure

All procedures were performed with uninterrupted or minimally interrupted novel oral anticoagulant therapy (1–2 doses held prior to procedure) or uninterrupted warfarin. Oral anticoagulants were resumed on the same day of the procedure. Up to three ultrasound‐guided femoral venous punctures were performed for each patient. Heparin boluses were administered to target an activating clotting time (ACT) of 350–400 s while catheters were in the heart. The energy source for ablation was selected at the discretion of the operator, with PFA (using 14F to 16.8F outer diameter sheath) supplanting CBA (with 14F to 15F outer diameter sheath) as the primary modality at these institutions in April 2024. Protamine sulfate was administered at the conclusion of each procedure.

### 2.3 Study Group (IM)

Each site was preclosed at the beginning of the procedure with a single SMVC device in each of the three femoral venous access sites. At the end of the case, the sheaths were removed, hemostasis was achieved by fastening the SMVCs without retaining a wire for access and brief manual pressure when needed. Successful closure was defined as immediate hemostasis without the need for prolonged manual pressure or supplementary closure devices, and the absence of clinically significant vascular complications. There was no period of mandated BR. The patients were permitted to move both legs immediately with no head of bed restriction. Patients could ambulate immediately at their choosing. Instructions were given to ambulate at 1 h if it had not already occurred. Discharge was protocolized at 3 h if the patient had met criteria for postanesthetic recovery without access site bleeding. The patients recovered in a recliner chair after the first hour of recovery when available. If access site bleeding occurred, manual pressure was held when needed and ambulation was repeated after additional recovery in the recliner chair.

### 2.4 Control Group (BR)

The right femoral vein was cannulated percutaneously by the modified Seldinger technique at three venotomy sites. At the conclusion of the procedure, hemostasis was achieved using a figure‐of‐eight nonabsorbable sutures and a three‐way stopcock, with manual compression. This was followed by a 4‐h flat BR and leg immobilization. Patients were discharged the same day if the criteria were met for vascular and postanesthetic recovery. If access site bleeding occurred, manual pressure was held when needed and BR was extended.

### 2.5 Postprocedural Care and Follow‐Up

Following the procedure, the access site was examined for vascular complications at a 2‐week follow‐up visit. Imaging studies and electronic medical charts were also reviewed to evaluate for any emergency department visits or hospitalizations related to the procedure.

### 2.6 Study Endpoints

The primary endpoints were clinically apparent vascular complications of bleeding that required additional intervention or transfusion, hematoma that required intervention, pseudoaneurysm, or vascular thrombus. The secondary endpoint was time to discharge, defined as the time from the end of the procedure to the time of discharge.

### 2.7 Statistical Analysis

We compared descriptive and clinical characteristics of patients between IM and BR groups using Student's *t*‐test or Wilcoxon rank sum test for continuous variables and *χ* ^2^ test or Fisher's exact test for categorical variables based on variable distribution. Continuous variables were presented as mean with standard deviation or median with interquartile range and categorical variables were presented as frequency with proportions. All *p* values are two‐tailed and < 0.05 were considered statistically significant. All statistical analysis was conducted using SAS, Version 9.4 (SAS Institute Inc., Cary, NC).

## 3 Results

### 3.1 Study Population

A total of 200 patients were retrospectively analyzed in this study, consisting of 100 consecutive patients in the IM group and 100 patients in the BR group. Hemostasis was attained in 100% of the IM group using SMVC. Baseline demographics were similar between the two groups with a mean age of 68.4 ± 11.7 years and a mean LVEF of 56.7% ± 11.9%. There were significant differences in clinical comorbidities of CAD, diabetes mellitus, and CHF, which were higher in the BR group (Table 1). In the IM group, 72% of the procedures were performed using PFA and the remaining using CBA, while in the BR group, 5% of the procedures were performed using PFA (Table 2).

**Table 1:** Baseline characteristics and comorbidities.

	Overall	IM	BR	
Baseline demographics	*n* = 200	*n* = 100	*n* = 100	*p* **value**
Age, years	68.3 ± 11.7	67.6 ± 12.8	69.0 ± 10.6	0.791
BMI, kg/m^2^	30.3 ± 7.1	30.1 ± 7.6	30.4 ± 6.6	0.541
LVEF, %	56.7 ± 11.9	58.4 ± 10.1	55.0 ± 13.4	0.286
Gender				0.198
Female, *n* (%)	85 (42.5)	47 (47)	38 (38)	
Male, *n* (%)	115 (57.5)	53 (53)	62 (62)	
CHA2DS2VASc	3 (2–4)	3 (1.5–4.0)	3 (2.0–4.0)	0.115
Oral anticoagulation				0.092
Apixaban, *n* (%)	163 (81.5)	86 (86)	77 (77)	
Rivaroxaban, *n* (%)	34 (17)	14 (14)	20 (20)	
Coumadin, *n* (%)	3 (1.5)	0 (0)	3 (3)	
Comorbidities				
Hypertension, *n* (%)	144 (72)	67 (67)	77 (77)	0.115
Coronary artery disease, *n* (%)	59 (29.5)	23 (23)	36 (36)	0.044
Stroke, *n* (%)	16 (8)	8 (8)	8 (8)	0.299
Diabetes mellitus, *n* (%)	54 (27)	17 (17)	37 (37)	0.001
Congestive heart failure, *n* (%)	56 (28)	21 (21)	35 (35)	0.028
Total procedure time, min	107.4 ± 36.9	117.7 ± 39.9	97.0 ± 30.5	< 0.0001
Total procedure time in minutes, range (42–273), median (IQR)	100.0 (81.0–123.0)	108.0 (93.5–134.0)	91.0 (75.0–109.5)	< 0.0001

**Table 2:** Procedural characteristics.

Type of procedure	Overall	IM	BR	*p* **value**
				< 0.0001
Cryoablation	123 (61.5)	28 (28)	95 (95)	
Pulse field ablation	77 (38.5)	72 (72)	5 (5)	

#### 3.1.1 Primary Endpoint

There was no significant difference in vascular complications between the two groups (1 IM vs. 0 BR, *p* = 0.316). In the IM group, one patient developed groin swelling. A 3 × 2 cm partially thrombosed pseudoaneurysm of the right common femoral artery was identified on ultrasound imaging. This resolved with no intervention on subsequent imaging performed a week later. In the BR group, there were no vascular complications (Table 3).

**Table 3:** Primary endpoint.

Vascular complications	Overall	IM	BR	*p* **value**
Groin hematoma	0 (0)	0 (0)	0 (0)	—
Bleeding	0 (0)	0 (0)	0 (0)	—
Pseudoaneurysm	1 (0.5)	1 (1)	0 (0)	0.316
Other	0 (0)	0 (0)	0 (0)	—

#### 3.1.2 Secondary Endpoint

Time to discharge was significantly shorter in the IM group (4.2 ± 2.6 h vs. 6.0 ± 2.7 h, *p* < 0.0001) (Table 4).

**Table 4:** Secondary endpoint.

Time to discharge	Overall	IM	BR	*p* **value**
Time to discharge, h	5.1 ± 2.8	4.2 ± 2.6	6.0 ± 2.7	< 0.0001
Time to discharge (h), range (0.6–23.4), median (IQR)	4.9 (3.6–5.6)	3.7 (3.4–4.2)	5.5 (5.2–5.9)	< 0.0001

## 4 Discussion

In this single‐center study, IM facilitated by SMVC was feasible following CA for AF using large access sheaths with no increase in the rate of vascular complications compared to BR. This strategy was also utilized as part of an earlier discharge protocol.

Vascular access remains the most common cause of complications following catheter‐based percutaneous electrophysiology procedures. Manual compression is the standard of care for hemostasis and remains the most prevalent modality [2]. Vascular closure devices (VCD) have been available in the USA since the 1990s and are being increasingly employed to improve safety, patient comfort, and facilitate same‐day discharge [5]. There are numerous approaches to device‐mediated vascular closure, namely the use of a collagen plug such, those that use clips and those that deploy suture‐mediated closure, such as Perclose (Abbott Vascular) [6]. Among the collagen‐mediated VCDs, the AMBULATE trial suggested that the VASCADE MVP VVCS is a safe and effective alternative to manual compression, offering enhanced recovery and patient comfort following multiple‐access CA procedures [3].

Many studies have evaluated the safety and efficacy of SMVC [6, 7]. Perclose Prostyle and ProGlide are SMVC devices that deliver percutaneous sutures enabling primary healing and remain the only devices marketed with the reported advantage of immediate mobilization following CA. PRO‐PVI study evaluated the feasibility of same day following radiofrequency ablation using SMVC [7]. Similarly, STYLE‐AF study demonstrated that the use of SMVC following CBA or PFA resulted in early time to discharge with no significant difference in vascular complications [8].

The current study builds upon prior literature by specifically evaluating a protocol of immediate mobilization of the legs, with no mandatory requirement for BR and no head of bed restriction. The patients recovered in a recliner chair after the first phase of recovery. If there were no vascular access site complications and postanesthetic criteria were met, they were discharged at 3 h postprocedure. The time to discharge in the IM group was significantly shorter compared to the BR group without affecting safety outcomes. These results are in line with the PRO‐PVI study, which found no vascular complications as well as the STYLE‐AF study which resulted in shorter time to ambulation and discharge with SMVCs [7, 8]. It is noteworthy that all vascular closure in this study were performed by highly experienced operators. The learning curve for delivering SMVC may limit the generalizability of the findings.

## 5 Limitations

We acknowledge the limitations of this study. First, this was a retrospective, nonrandomized study conducted at a single health system, and different proportions of cases were performed by different operators between the two groups which may limit the generalizability of the findings. Additionally, while the endpoints were vascular complications and time to discharge, we anticipate that the most meaningful impact of IM may be an improvement in patient comfort and satisfaction, which were not studied. Another limitation is the absence of a detailed cost analysis. It is possible that earlier discharge, a reduction in staff utilization for access site management, and decompression of recovery areas may, in part, offset the cost of SMVC devices. Future studies evaluating clinical and patient‐centered outcomes, as well as the economic impact of IM would be valuable in determining the broader implications of adopting immediate mobilization in clinical practice.

## 6 Conclusions

In this single‐center study, IM facilitated by SMVC following CA for AF using large access sheaths did not increase the rate of vascular complications compared to a 4‐h BR protocol and resulted in a shorter time to discharge. Further studies are needed to illustrate the benefits to patient comfort and satisfaction that potentially result from this approach.

## Ethics Statement

This study was approved by the Endeavor Health – NorthShore University Institutional Review Board with waiver of consent.

## Conflicts of Interest

Jeremiah Wasserlauf MD, MS, FHRS: Boston Scientific (research support), Sonothera (consulting), Atraverse (consulting).

## Data Availability

The data that support the findings of this study are available in the Supporting Information of this article.
